# Pattern of Orofacial Clefts at A Tertiary Care Hospital in Ethiopia

**DOI:** 10.4314/ejhs.v31i6.12

**Published:** 2021-11

**Authors:** Mekonen Eshete

**Affiliations:** 1 Consultant Plastic and Reconstructive Surgeon Surgical Department, School of Medicine, College of Health Sciences Addis Ababa University; Members of the Smile Train Research & Innovation Advisory Council; Head Yekatit 12 Hospital Medical College Plastic and Reconstructive unit

**Keywords:** Orofacial clefts, Patterns, Ethiopia

## Abstract

**Background:**

Clefts of the lip and/or palate are the most common craniofacial birth defects. The worldwide birth prevalence is 1/700 live births. There are varying reports from Africa. This study investigated the patterns of orofacial clefts at a tertiary care hospital in Addis Ababa.

**Methods:**

A retrospective descriptive study was performed to assess the patterns of Orofacial clefts at the main cleft care center in Ethiopia. The Data of cleft patients operated at the main cleft care center in Ethiopia from January 2007 to April 2020 with the support of Smile Train was used for this study. Their demographic and clinical data was retrieved from the Smile Train data base and analyzed using Stata version 16.

**Results:**

A total of 1919 patients' data was retrieved, excluding 16 patients' data (.83%). The data of 1903 (99.17%) patients were enrolled in this study. Cleft lip and palate were found in 53.0% of the patients. Cleft lip only was found in 731 (38.4%) and cleft palate only in 166 (8.6%) patients. The commonest surgery performed was primary unilateral lip nose repair. Most patients were operated after the age of five years old.

**Conclusion:**

Many were operated after the age of five years, which is not in line with international recommendations. This needs improvement: establish more cleft care centers, distribute health care information and education.

## Introduction

Ethiopia is the second most populous country in sub-Saharan Africa with a population of 112,078,730 as of 2019 and estimated by the United Nations to reach 205,410,671 by 2050. (1) Currently, Ethiopia is divided into 10 regions. Oromia is the largest region occupying 34 percent of the land area. The highest concentration of Ethiopians (37%) live in the Oromia region followed by Amhara region where 22% of Ethiopians live. (2, 3).

The majority of Ethiopian population (78.78%) lives in a rural setting (4). In the Ethiopian health care system subspecialty health care such as plastic and reconstructive surgery is still at its infancy. The care of children born with birth defects is neglected and there is neither a neonatal screening program nor a birth defect registry system. The government's health care priority is in tackling infectious diseases like tuberculosis, malaria, and HIV/AIDS. According to the World Health Organization (WHO), infant mortality associated with birth defects, including orofacial clefts, is increasing worldwide. (5) The contribution of birth defects to infant mortality in Ethiopia is currently unknown because of the lack of a registry and study.

Until 2003, no standardized cleft care program existed in Ethiopia. In 2003, a cleft care center was established at one of the public hospitals with the support of the Norwegian Government. This center was strengthened slowly, and subsequently in 2007 began collaborating with Smile Train and Transforming Faces, both charity organizations which support free cleft care. This center also established regional and other international collaborations to strengthen cleft care at the unit and other hospitals in the country.

The collaboration with Smile Train, the world's largest cleft charity, not only supported us in providing safe and standardized cleft care, but also provided an electronic system to keep records of operated patients in an online data base. Smile Train Express (STX), Smile Train's electronic records system, has great importance in the assessment of surgical care and in conducting research especially when there is no birth defect registry system in place. We used STX to analyze the patterns of orofacial clefts at the main cleft care center in Ethiopia.

Orofacial clefts (OFCs) occur in the most visible part of a human body subjecting affected individuals to heavy stigmatization. They are the commonest birth defects in the head and neck region and affect 1 in 700 live births worldwide (6). The prevalence reported varies from 1/2500 to 1/500 births depending on the geographic region, racial and ethnic backgrounds, and socioeconomic status (7, 8). This difference in prevalence across the world indicates the role of multiple genetic and environmental factors in the occurrence of OFCs. In Ethiopia, the exact number of people with orofacial clefts is unknown because of inexistent birth defect registry systems and absent national surveying of birth defects inclusive of OFCs. A 2011 study done at Addis Ababa, Ethiopia health institutions revealed an OFC incidence of 1.49 per 1000 live births (9). Another Ethiopian retrospective hospital-based study found an OFC incidence of 44 per 100,000 live births and a prevalence of 20 per 100,000 population (10). The incidence, prevalence, and distribution of OFCs in other African countries have been previously investigated (11–17). The majority of studies done in Africa including Ethiopia showed that a low prevalence, however most of the studies were hospital based with a small sample size. (18) This might be the reason for the lower estimate of the prevalence. In Ethiopia and many other African countries, a significant proportion of births take place at home where there is no recording. This may have also contributed to lower estimates from some countries.

As already mentioned, there is no birth defect registry system in Ethiopia and few hospital-based studies have been done on the incidence and prevalence of general birth defects or OFC defects in particular. This study assessed the data of 1919 patients born with orofacial clefts operated with the support of Smile Train at the main cleft care center in Ethiopia. It is the only center where multidisciplinary cleft care is provided and includes speech therapy services as well.

## Methods

The data of all operated patients at our Smile Train supported center was prospectively entered into STX, Smile Train's patient database. Ethical clearance was obtained from the Institutional Review Board at Yekatit 12 Hospital Medical College, Addis Ababa – Protocol number 61/20. Smile Train also approved the use of the data. We retrieved data of all new Smile Train supported cleft patients operated at the main cleft center in Ethiopia, which included data of all cleft patients receiving single surgical treatment between March 2007 and April 2020. We assessed the following variables: type of cleft, cleft laterality, gender, age at operation, and address of the patients. All individuals receiving rehabilitative surgery with the support of other charity organizations were not included. Syndromic and atypical clefts were also not included in this study.

Frequency tables were constructed for the overall sample and stratified by gender. Exact binomial tests for differences in proportions were used for the whole population and for each gender to assess whether there was a significant difference in the proportion of bilateral cleft lip and palate (BCLP), left/right cleft lip and palate (CLP), and unilateral cleft lip and palate (UCLP). An analogous procedure was followed for the patients with cleft lip only (CLO). Information about immediate and distant relatives with clefts was also collected.

## Results

After obtaining ethical clearance from the Yekatit 12 Hospital Medical College and with permission from Smile Train data from STX was accessed, resulting in a total of 1919 patient. The diagnoses of 16 patients (0.83%) were not complete and thus excluded from analysis. The data of 1903 (99.17%) patients was found to be complete and included in the analysis. There were 1194 (62.7%) males and 709 (37.3%) females, resulting in a male to female ratio of 1.7:1.

The largest portion of patients were from the Oromia region at 823 (43.2%), followed by Addis Ababa 560 (29.4%); 311 (16.3%) were from Amhara region, 149 (7.8%) were from Southern Nations Nationalist and Peoples' region (SNNPR), 60 (3.2%) were from other regions (Tigray, Harari, Dire Dawa, Benishangul-Gumuz, and Somali).

The distribution of orofacial clefts found in this study was cleft lip only at 731 (38.4%), cleft lip and palate 1006 (52.9%), and cleft palate only 166 (8.7%). Both cleft lip only and cleft lip and palate was found more commonly in males with a ratio of 1.53 and 2.02; respectively. Isolated cleft palate was found more commonly in females with a ratio of 0.89.

Cleft laterality was also assessed. The majority of CLO occurred on the left side with 403 cases (21.18%), followed by right side CLO with 208 cases (10.93%), and BCLO at 120 (6.31%). From the cleft lip with cleft palate group, bilateral cleft lip and palate (BCLP) was the most common with 396 (20.81%) followed by left side CLP with 384(20.18%) (Table 1). Table 1 also shows an estimate of the expected frequency for males and female under the null hypothesis.

**Table 1:** The distribution into the various cleft types

Frequency	BCLP	BCLO	CPO	LCLO	LCLP	RCLO	RCLP
Female	123	49	86	165	129	75	82
Female expected	147.5	44.7	61.8	150.9	143.1	77.5	84.2
Male	273	71	80	238	255	133	144
Male expected	248.5	75.3	104.2	252.9	240.9	130.5	141.8
Total	396	120	166	403	384	208	226
Percent	20.81	6.31	8.72	21.18	20.18	10.93	11.88

Table 2 shows the frequencies for the unilateral and bilateral cleft lip and palate and cleft lip only, followed by the test results. The binomial test showed that the proportion of unilateral cleft lip and palate is greater than bilateral cleft lip and palate (p<0.0001). Similarly, the proportion of unilateral cleft lip only is greater than bilateral cleft lip only (p<0.0001).

**Table 2:** Distribution and comparison between individuals with unilateral and bilateral cleft lip and palate and cleft lip only

	Cleft lip and palate			

	Frequency	Percent	Frequency of females	Frequency of males
Bilateral	396	39.36	123	273
Unilateral	610	60.64	211	399
Total	1,006	100	334	672
	**BCLP percent**	**Confidence Interval**		**Exact p-value**
Bilateral vs Unilateral	39.36	(36.39, 42.42)		<0.0001
	**Cleft lip only**			
Bilateral	120	16.42	49	71
Unilateral	611	83.58	240	371
Total	731	100	289	442
	**BCLP percent**	**Confidence Interval**		**Exact p-value**
Bilateral vs Unilateral	16.42	(130.90, 19.29)		<0.0001

As seen in Table 3 there is no difference in the proportion of bilateral cleft lip and palate and left side cleft lip and palate (p-value 0.6674). The proportion of bilateral cleft lip and palate is greater than the proportion of right-side cleft lip and palate (p-value 0.0001). The proportion of right-side cleft lip and palate is less than the proportion of left side cleft lip and palate (p-value 0.0001). Similarly, the proportion of bilateral cleft lip only is less than the proportion of left side and right-side cleft lip only (p-value 0.0001). The proportion of left side cleft lip only is greater than the proportion of right-side cleft lip only (p-value 0.0001).

**Table 3:** Distribution and comparison between left versus right and between each side versus bilateral cleft lip and palate

	Frequency Percent	Frequency of females		Frequency of males
**Bilateral**	396	39.36	123	273
**Left**	384	38.17	129	255
**Right**	226	22.47	82	144
**Total**	1006	100	334	672
		**Percent of first category** **interval**	**Confidence**	**Exact p-value**
**Bilateral vs Left**		50.77	(47.26, 52.74)	<0.6674
**Bilateral vs Right**		63.67	(59.00, 67.36)	<0.0001
**Left vs Right**		62.95	(59.4, 66.7)	<0.0001
	Distribution and comparison between left versus right and between each side versus bilateral cleft lip only			
	**Frequency** **Percent**	**Frequency of** **females**		**Frequency of males**
**Bilateral**	120	16.42	49	71
**Left**	403	55.13	165	238
**Right**	208	28.45	75	133
**Total**	731	100	289	442
		**Percent of first category** **interval**	**Confidence**	**Exact p-value**
**Bilateral vs Left**		22.94	(19.53, 26.75)	<0.0001
**Bilateral vs Right**		36.59	(31.53, 41.96)	<0.0001
**Left vs Right**		65.96	(62.10, 69.62)	<0.0001

A similar analysis was done but stratified by gender (Table 4). Among females, there is no significant difference in the proportion of bilateral cleft lip and palate and left side cleft lip and palate (p-value 0.7055). The proportion of right-side cleft lip and palate is less than the proportion of bilateral cleft lip and palate and left side cleft lip and palate (p-value <0.0042 and 0.0012 respectively). Similarly, in males there is no significant difference in the proportion of bilateral cleft lip and palate and left side cleft lip and palate (p-value 0.4334). The proportion of right-side cleft lip and palate is less than the proportion of bilateral cleft lip and palate and left-side cleft lip and palate (p-value 0.0001 in both cases) table 5. In this study the proportion of left-side cleft lip only in females is greater than the proportion of bilateral cleft lip only and right-side cleft lip only (p-value in both cases <0.0001). The proportion of right-side cleft lip only is greater than the proportion of bilateral cleft lip only (p value 0.0196). In males, the proportion of left-side cleft lip only is greater than the proportion of bilateral and right-side cleft lip only (p-value <0.0001). The proportion of right-side cleft lip only is higher than the proportion of bilateral cleft lip only (p-value <0.0001). There is no significant sex difference in cleft palate only (p-value 0.6414).

**Table 4:** Distribution and comparison between females and males with left versus right and between each side versus bilateral cleft lip and palate and cleft lip only

Females (cleft lip and palate)			

	Frequency	Percent	
**Bilateral**	123	36.83	
**Left**	129	38.62	
**Right**	82	24.55	
**Total**	334	100	
	**Percent of first category**	**Confidence interval**	**Exact p-value**
**Bilateral vs Left**	75.43	(71.55, 78.93)	<0.7055
**Bilateral vs Right**	82.85	(79.19, 85.97)	<0.0042
**Left vs Right**	82.40	(78.67, 85.61)	0.0012
**Males (cleft lip and palate)**			
	**Frequency**	**Percent**	
**Bilateral**	273	40.62	
**Left**	255	37.95	
**Right**	144	21.43	
**Total**	672	100	
	**Percent of first category**	**Confidence interval**	**Exact p-value**
**Bilateral vs Left**	60.83	(57.02, 64.52)	<0.4334
**Bilateral vs Right**	73.33	(69.43, 76.90)	<0.0001
**Left vs Right**	72.73	(68.76, 76.36)	<0.0001
**Females (cleft lip only)**			
	**Frequency**	**Percent**	
**Bilateral**	49	16.96	
**Left**	165	57.09	
**Right**	75	25.95	
**Total**	289	100	
	**Percent of first category**	**Confidence interval**	**Exact p-value**
**Bilateral vs Left**	42.11	(36.48, 47.94)	<0.0001
**Bilateral vs Right**	61.54	(54.48, 68.14)	0.0196
**Left vs Right**	84.31	(80.76, 87.31)	<0.0001
**Males (cleft lip only)**			
	**Frequency**	**Percent**	
**Bilateral**	71	16.06	
**Left**	238	53.85	
**Right**	133	30.09	
**Total**	442	100	
	**Percent of first category**	**Confidence interval**	**Exact p-value**
**Bilateral vs Left**	33.52	(28.80, 38.59)	<0.0001
**Bilateral vs Right**	47.43	(41.32, 53.62)	<0.0001
**Left vs Right**	75.19	(71.34, 78.67)	<0.0001
**CPO**			
**Males**	80	40.64, 55.83	0.6414
**Females**	86		

**Figure 1:**
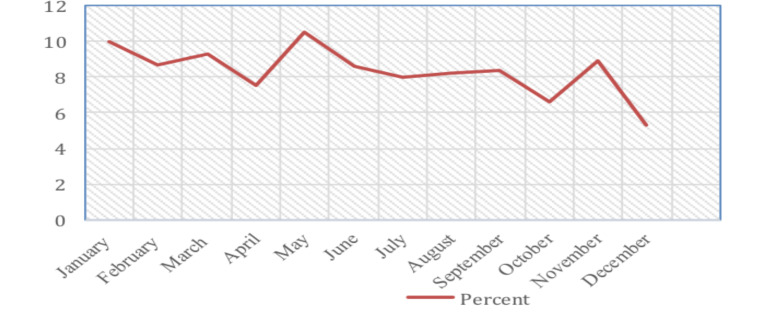
This figure shows that the birth of children with clefts is higher in the month of May and least in the month of December.

This study showed that the greatest number of cleft patients were born in the month of May (10.5%) and the fewest were born in December (5.2%) Figure 1.

In this study, few patients gave history of clefts in their family; 35 (1.8%) patients gave history of clefts in immediate relatives and 37 (1.9%) gave history of clefts in other relatives.

The type of surgical procedure performed was also assessed. The most common surgery performed was primary unilateral lip/nose repair (51%), followed by primary cleft palate repair (27%). Lip nose revision was performed for 135 (7%) of cases. Very few rehabilitative surgeries such as alveolar bone graft, secondary rhinoplasty, etc. were performed.

The majority of the patients (803) were operated on after the age of 5 years old. Of the 1903 patients, only 395 (20.76%) received surgery at the age of one year old or less. Palatoplasty was done for 512 patients; of these only 32 (6.3%) were operated at the age of one year old or less.

For geographical location and age at surgery, 40% of the patients who were operated at the age of one year or less were from Addis Ababa; 32% were from Oromia, 14.8% were from Amhara region, and the rest were from SNNPR and other regions. Of those who were operated after the age of 18 years old, 49.6% were from Oromia region and 23.2% were from Addis Ababa.

**Figure 2:**
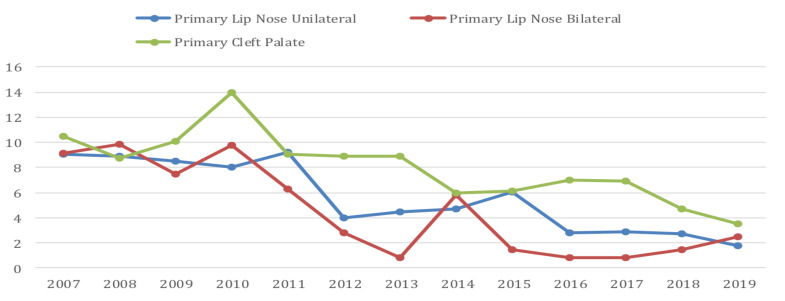
Average age at the time of surgery for different types of clefts

The average age for unilateral cleft lip surgery was 9.08 years; for bilateral cleft lip surgery was 9.17 years and for cleft palate surgery was 10.5 years in 2007. By 2019, the average age for surgery decreased significantly at our unit to 1.75 years for primary unilateral cleft lip; 2.5 years for bilateral cleft lip and 3.5 years for palate repair (Figure 2).

## Discussion

The cleft center at Yekatit 12 Hospital Medical College is the main cleft center Ethiopia and providing both primary and secondary cleft surgeries. It was established in 2003 with the support of the Norwegian Government. Currently almost all primary cleft surgeries at this center are done in partnership with Smile Train, with the patient data being recorded in Smile Train Express (STX) prospectively. This study was done using the STX database. In the absence of a birth defect registry system and community-based studies, using well organized database such as STX is an excellent alternative.

This study analyzed the post-surgical data of patients born with orofacial clefts, operated at Yekatit 12 Hospital Medical College cleft care center in partnership with Smile Train. The data of the operated patients was retrieved from the charity organization's electronic health records system, STX. The data was then analyzed by gender, geographic location, cleft type, laterality, age at the time of surgery, and type of surgery.

A large portion of the patients (43.2%) were from the Oromia region. This is probably because of geographical vicinity since our center is situated in Addis Ababa, surrounded by the Oromia region. The other reason could simply be because of the region's population size, as Oromia is the most populous region in Ethiopia containing 34.4% of the population. In this study 560 (29.4%) patients were from Addis Ababa; we conclude this is because of vicinity to our center. 311 (16.3%) of the cleft patients in this study were from Amhara Region; the most probable reason could be the region's population size as it contains the second largest population of Ethiopians. 149 (7.8%) were from Southern Nations Nationalist People's Region, which is the third most populous region in Ethiopia. 60 (3.2%) were from other regions (Tigray, Harari, Dire Dawa, Benishangul-Gumuz, and Somali).

The distribution of cleft types was assessed for 1903 patients and showed that cleft lip and palate was the most common, with 1006 (53.0%) followed by cleft lip only 731(38.4%), this is similar to a study done using the same data source but includes other sub-Saharan countries as well as Ethiopia (11). The result is also similar to another prospective study done in Addis Ababa from 2011 (19). Jensen et al observed that CLP is twice as common as CLO (20). One African study done by Butali et al reported more CLO cases, followed by CLP cases, it is also different from a previous Ethiopian study which showed that CLO was the majority followed by CLP (21). This is explained because this study captured data of patients operated at the main cleft care center in the country where more severe clefts were referred. CPO is less in this study in which is comparative to many other African studies(12) (11); CPO is also slightly more in females with a male to female ratio of 0.89.

In this study all types of clefts are more common in males than in females, with the exception of isolated CP. Another Ethiopian study using STX included data of all Ethiopian cleft patients operated at 31 Ethiopian hospitals. It showed that all types of clefts including isolated cleft palate are more common in males (22). A 2009 study done by Mossey et al reported that non-syndromic cleft lip and palate is twice as common in males than in females while cleft palate only is twice as common in females than in males (23).

The largest portion of the cleft patients were born in the month of May (10.5%), with the least in December. This may be explained by the preconception months (June, July, August and September) being the most difficult months in most parts of Ethiopia. During these months there is greater food scarcity and increased workload on the female population as it is ploughing time. Additionally, the rainy season involves increased contact with chemicals and fertilizers but this needs further prospective case control study. There is evidence that maternal environment, malnutrition and vitamin deficiency especially folic acid contributes to the occurrence of orofacial clefts and maternal malnutrition varies seasonally (24, 25). A retrospective study by Rachael et al (26)reported seasonal variation in the occurrence of cleft lip and palate in Zambia. In this study more children with cleft lip and palate were born in March through August than in September through February. They concluded that this could be explained in part by environmental factors. Another African retrospective study by Sofianos et al (27) reported seasonal variation in the incidence of orofacial clefts limited to patients with both a CL/P. In this study more children were born with a CL/P in winter months. The reason they gave was sunlight exposure and vitamin D levels, weight gain, and maternal obesity. A case control retrospective study by Miroslav Peterka et al (28) found significant differences from controls in the number of new-born girls with CL and boys with CP, whose dates of birth correspond to conception from April to August and to the estimated prenatal critical period for cleft formation from May to October. The reason they gave was that the season May to October is a warm period and during this period various injurious physical, chemical and biological factors may act on a pregnant woman and contribute to the occurrence of the anomaly.

The greatest portion of patients receiving surgery at the age of one year old or younger were from Addis Ababa. This may be explained because of awareness about the treatability of this anomaly and vicinity making access to treatment easier.

The average age at surgery in 2007 and 2019 was different and decreasing significantly over time. This is a clear indication that our center's learning curve improved over that time period. In 2007 the cleft unit at the study institution began collaborating with Smile Train and Transforming Faces, both supporting free cleft care and training at this unit. Expert cleft surgeons from all over the world were invited to conduct various short courses on cleft surgery collaborating and improving our local education and skills. This contributed to improved expertise and confidence levels for our cleft center's surgical team. We believe this is the main reason for reducing the average age for surgery. Another possible contributing factor to the reduction of the average age for surgery could be that the backlog of unoperated cleft patients is reduced, with intake of mainly new cases. At the same time, increases in information dissemination regarding the treatability of this defect may be a cause for families to bring their children for treatment at earlier age.

The surgery most commonly performed was primary unilateral lip/nose repair (50.7%), a similar finding to previous studies. The second most common surgery was primary cleft palate repair with 512 cases (27.7%). This differs from the study done by Conway et al (11) and Butali et al (12). Of the 512 patients operated for palate surgery, only 32 (6.25%) were operated at the age of 1 year old and younger. According to our speech therapists, the majority of palatoplasty patients receiving speech therapy had intelligible speech despite palatoplasty being performed at later ages for the majority. This contradicts studies which suggest that palatoplasty done at early age contributes to the development of near normal speech development (29) (30–33). Our study reveals that very few rehabilitative surgeries like (alveolar bone graft, secondary rhinoplasty, etc.) were performed at our center.

Most of the patients in this study were operated after the age of five years old. This has an obvious effect on the outcomes of surgery, especially when considering speech development and the huge psychological impact on those operated later in life. One of the reasons for not providing timely surgery is the vast backlog of cleft cases at our center and in our country. Therefore, we recommend establishing more multidisciplinary cleft care centers in Ethiopia. We used hospital-based data for this study which is prone to selection bias. As such, we further recommend population-based studies and establishing a birth defect registry system to gain further insight in the challenges faced by our country in the management of orofacial clefts

The data for this study comes from one of the few multidisciplinary cleft care providing centers in Eastern Africa therefore it provides a relevant information about the patterns of orofacial clefts and will serve as reference for future population-based studies. However, it is limited by using a single hospital's data and may not be representative of the true picture of the distribution of clefts in our country and in the Eastern part of our continent.
